# The Survey of Cells Responsible for Heterotopic Ossification Development in Skeletal Muscles—Human and Mouse Models

**DOI:** 10.3390/cells9061324

**Published:** 2020-05-26

**Authors:** Łukasz Pulik, Bartosz Mierzejewski, Maria A. Ciemerych, Edyta Brzóska, Paweł Łęgosz

**Affiliations:** 1 Department of Orthopaedics and Traumatology, Medical University of Warsaw, Lindley 4 St, 02-005 Warsaw, Poland; lukasz.pulik@wum.edu.pl; 2 Department of Cytology, Faculty of Biology, University of Warsaw, Miecznikowa 1 St, 02-096 Warsaw, Poland; bmierzejewski@biol.uw.edu.pl (B.M.); ciemerych@biol.uw.edu.pl (M.A.C.)

**Keywords:** muscles, heterotopic ossification, skeletal muscle stem and progenitor cells, HO precursors

## Abstract

Heterotopic ossification (HO) manifests as bone development in the skeletal muscles and surrounding soft tissues. It can be caused by injury, surgery, or may have a genetic background. In each case, its development might differ, and depending on the age, sex, and patient’s conditions, it could lead to a more or a less severe outcome. In the case of the injury or surgery provoked ossification development, it could be, to some extent, prevented by treatments. As far as genetic disorders are concerned, such prevention approaches are highly limited. Many lines of evidence point to the inflammatory process and abnormalities in the bone morphogenetic factor signaling pathway as the molecular and cellular backgrounds for HO development. However, the clear targets allowing the design of treatments preventing or lowering HO have not been identified yet. In this review, we summarize current knowledge on HO types, its symptoms, and possible ways of prevention and treatment. We also describe the molecules and cells in which abnormal function could lead to HO development. We emphasize the studies involving animal models of HO as being of great importance for understanding and future designing of the tools to counteract this pathology.

## 1. Introduction

Heterotopic ossification (HO) is a disregulation of skeletal muscle homeostasis and regeneration, which results in mature bone formation in atypical locations. HO could develop in the skeletal muscles, and also in surrounding tissues such as fascia, tendons, skin, and subcutis [1]. HO can be acquired or have genetic origin. The most prevalent is acquired HO which can occur in response to a direct trauma, burn, or amputations. Similarly, iatrogenic trauma, caused by orthopedic surgery such as hip replacement, often triggers HO development [1,2]. Another acquired form of the disease is neurogenic HO (NHO) which is a frequent complication of central nervous system injury [3]. The knowledge about the molecular mechanisms that leads to HO formation and cell precursors engaged in this process is still limited. HO requires the presence of stem or progenitor cells which are able to follow the osteogenic program, although the identity of these cells remains unclear. Many different populations of progenitor cells could be possible precursors in the HO development. The animal studies suggest that progenitor cells can vary depending on the HO subtype. The studies using mouse HO models show that endothelial cells, mesenchymal cells, pericytes present in the skeletal muscles, tendons and connective tissue cells, or even circulating stem/precursor cells could be a source of HO precursors [1,4,5]. It is also known, that trauma or micro-trauma, which leads to a local inflammatory response, delivers the signals to develop HO. Recent studies showed the role of immune response cells, especially monocytes/macrophages, at the early stages of trauma-induced HO development [6]. They confirm the importance of macrophages in the induction of neurogenic and genetic forms of HO [7]. Activated macrophages express osteoinductive signaling factors in the course of HO pathogenesis. Thus, the presence of the cells reflects increased secretion of HO promoting cytokines/chemokines such as interleukin 6 (IL6), IL10, transforming beta-1 growth factor (TGFβ1), and neurotrophin 3 (NT3). A significant dysregulation of macrophage immune checkpoints was proven in HO animal models [8,9,10]. Finally, both individual predisposition and risk factors also attribute to HO development [11].

Histologically HO formation is similar to the physiological bone fracture healing. During HO development initially soft tissue is infiltrated with the whole spectrum of inflammatory cells. Such infiltration is followed by enhanced fibroblast proliferation, neovascularization, differentiation of chondrocytes, and results in mature bone formation [12]. HO is formed mainly by endochondral ossification. However, an intramembranous mechanism can also be involved. Typically HO formation is characterized by a zonal bone development model called “eggshell calcification” [13]. HO consists mainly of mechanically weak woven bone with an irregular osteoblasts distribution, but mature lamellar bone with Haversian-like canals can be often found. The bone tissue gradually matures with the outer appearance of the cortical bone [14]. Primarily, HO occurs in soft tissues and has no connection with the skeletal bone, but when it grows in the volume, it can attach to the periosteum.

## 2. Heterotopic Ossification as a Clinical Issue

HO is a diverse pathologic process and its spectrum can range from mild, clinically irrelevant to severe cases. In most of the patients it is minor and symptomless. Unfortunately, in some patients, extensive HO located around joints can cause restrictions in the range of motion (ROM), resulting even in the total ankylosis of the joint. In this group of patients HO can be associated with a significant limitation of daily activities and disability [1].

### 2.1. Traumatic HO

HO lesions can occur in response to direct trauma, such as connective tissue injury, bone fractures, burns, amputations, and combat-related blast injuries [2]. Approximately 30% of all fractures and dislocations which were subjected to operative treatment can trigger HO formation. It was a recognized clinical problem, in the acetabular and proximal femur fractures and fractures or dislocations of the elbow [1,15]. HO is also a common complication of traumatic limb amputation, both in civilian (22.8%) and in a military setting (62.9%) [16]. After the isolated burn injury HO incidence is relatively low (1%–4%), but it can be underestimated due to the lack of routine x-ray screening of such patients [17,18]. The typical locations of burn-induced HO are the elbow (50.0%), glenohumeral joint (20.3%), and the hip joint (17.6%) [18].

Recognized risk factors for trauma-induced HO are young age, male sex, severe concomitant injuries, compound fractures, extensive surgical approaches, and postponed surgeries [19,20]. The presence of local wound infection is also a well-established factor associated with HO [21]. In the military setting, the high incidence of HO is associated with concomitant brain injury, multiple wounds, and the severity of the injury [2]. The extent of burns, local wound infection, and the duration of intensive therapy are the risk factors for the formation of burn-induced HO. The role of immobilization and iatrogenic paralysis is also under investigation [18,22].

As far as the symptoms are concerned it can be associated with reduced joint mobility, pain, and decreased limb function. In the upper extremity, HO can limit everyday activities such as eating, dressing, and personal grooming, while in the lower limb it can affect gait and cause limp and difficulties in sitting [15]. Patients with amputation associated HO may experience difficulties with prosthesis fitting. Other local complications can occur, such as ulcers, skin graft necrosis, and neurovascular impairment [16]. The contractures and reduction of joint ROM in burn injuries are often caused by soft tissue scarring. However, HO should always be taken into consideration in differential diagnosis [18].

### 2.2. Surgery-Induced HO

HO is a well-described complication of orthopedic surgical procedures, typically joint replacements. The main indication for this kind of treatment is symptomatic, end-stage joint osteoarthritis. This type of HO usually involves the tissues where the surgical approach is performed, as unavoidable trauma is done to the muscle and fascia. It is most commonly described after a total hip replacement (THR) and cervical total disc arthroplasty (CTDA). It was also reported after the ankle, knee, and shoulder arthroplasty [23,24,25]. HO is radiologically present in every second patient after total hip replacement (40%–56%), and cervical total disc arthroplasty (44.6%–58.2%). However, high-grade HO occurs only in 2%–7% of total hip replacement and 11%–16% of cervical total disc arthroplasty patients [24,25,26].

The risk factors for the development of HO in patients undergoing total hip replacement are young age and male sex. The other predisposing conditions are bone and joint diseases such as ankylosing spondylitis, hypertrophic arthritis, and Paget’s disease. The impact of the surgical approach, especially micro-invasive surgery (MIS) techniques, is being intensively discussed, but the results are still inconclusive [24,27,28]. Similarly, in patients after the cervical total disc arthroplasty, the male sex is an independent risk factor for HO development. Another important aspect is an artificial disc device type [29].

In the majority of patients suffering from surgery induced HO, small islands of the bone of no clinical significance are observed. However, extensive lesions can affect the biomechanical function of an endoprosthesis and block the movement in the affected joint. In total hip replacement patients, high-grade HO can significantly impact ROM, especially flexion, abduction, and external rotation, and affect the overall function of the hip [30]. In extreme cases, HO can require surgical intervention and excision (3.3%) [27]. In contrast to total hip replacement, in cervical total disc arthroplasty patients, severe HO does not affect patient-related pain, quality of life, or function [31].

### 2.3. Neurogenic HO

Neurogenic heterotopic ossification (NHO) can occur after the spinal cord injury (SCI) or traumatic brain injury (TBI). Other clinical conditions, such as cerebral stroke, anoxia, and non-traumatic myelopathies, can also attribute to NHO [32,33,34]. The incidence of NHO in spinal cord injury (40%–50%) was reported to be higher than in traumatic brain injury patients (8%–23%). However, symptomatic NHO is more frequent in traumatic brain injury than in spinal cord injury patients (11% vs. 4%). The incidence of this type of HO in cerebral stroke patients is relatively low (0.5%–1.2%) [35,36]. In contrast to traumatic lesions, NHO lesions typically occur in locations distant from the site of injury. The NHO lesions are usually located around the hip joints in both spinal cord injury (63%) and traumatic brain injury (40%) [36]. Other possible locations of NHO are the shoulder, knee, and elbow joints [37,38].

The demographic risk factors predisposing to NHO after spinal cord injury are male sex and young age. The complete spinal cord injury, high level of rupture, and spasticity can also be associated with an elevated incidence of NHO. Moreover, urinary tract infections and pneumonia significantly increase the risk of NHO [1,38]. NHO may also be associated with human leukocyte antigen B27 (HLA-B27) presence in spinal cord injury patients [39]. In traumatic brain injury patients, the NHO associated conditions are lower walking abilities, spasticity, pressure ulcers, neurogenic bladder, and systemic infections [40].

NHO usually develops two months after a spinal cord injury or traumatic brain injury [36,41,42]. Initially, it is characterized by inflammation-like prodromal symptoms such as swelling, redness of the joint, and low-grade fever. If there is no sensory impairment, the pain can also be present. Usually, two years after the neurological event, the lesions are fully developed [3,36]. Most of the patients do not suffer from NHO associated symptoms. However, when the lesions are extensive, it can affect joint ROM creating problems with moving from sitting to lying position and nursing. Additionally, the risk of bedsores significantly rises [3,37]. Moreover, patients with severe NHO obtain less satisfactory functional results and require prolonged rehabilitation [43].

### 2.4. Genetic HO

There are also rare, inherited forms of HO, such as fibrodysplasia ossificans progressiva (FOP) and progressive osseous heteroplasia (POH) [44,45]. FOP is autosomal-dominant disorder caused by up to 14 different mutations localised in the type I bone morphogenic protein (BMP) receptor, i.e., activin type 1 receptor (ACVR1; also called activin-like kinase 2, ALK2) gene [44,46]. However, a single mutation, i.e., arginine to histidine at position 206; R206H, is present in the majority of FOP patients [44,46]. The ossification of skeletal muscles in FOP occurs mostly in early childhood and is characterized by inflammation-like symptoms and episodical flareups [44]. POH is the other autosomal dominant inherited form of HO, which is caused by the mutation in gene encoding guanine nucleotide-binding protein, alpha stimulating (GNAS) [47]. The exact incidence of FOP is estimated to be approximately 1 person per 2 million [47]. The epidemiological data of 299 FOP patients from fifty-four countries participating in the International FOP Association (IFOPA) Global Registry will be published soon [48]. In most cases of FOP, it is caused by de novo mutations, but there is also a risk of parental transmission [49]. The incidence of POH is unknown. Similarly to FOP, in POH family transmissions have been documented, but the majority of the patients have spontaneous mutations [47]. There are no identified predisposing factors for inherited HO, including ethnic, racial, or geographic factors [47,50].

Children suffering from FOP are born with characteristic deformities of the toe and then, usually between 5 and 10 years of age, start to present soft tissue swelling which could be spontaneous or caused by minor injuries. With age, mature bone appears at the site of edema in the muscles and surrounding tissues. HO can appear in any location except for the viscera and thoracic diaphragm. The first affected areas are neck and upper back. Progression of HO over time leads to mobility restriction, respiratory problems, and heart failure associated with intercostal and spinal muscle ossification and chest deformation [50]. Recent studies revealed dysmorphology of the hip, spine, and tibiofibular joint, which can predispose to the high incidence of arthropathy in FOP patients [51]. Other aspects of FOP are malnutrition due to temporomandibular joint ossification and hearing problems due to middle ear HO. Most patients use a wheelchair at the end of their second decade of life [47,49]. In contrast to FOP, the HO lesions in POH usually appear early, i.e., during the first year of life. POH starts from the skin and subcutis and later on affects the deeper-lying striated muscles and fascia. POH is characterized by changeable expressivity and somatic mosaicism, including asymptomatic carriers. In some cases with high expression, it can result in early severe disability with joint ankylosis. The sings of POH can also include growth retardation, osteoporosis, and low body weight. The diagnostic criteria for POH have been proposed [45].

### 2.5. Diagnostic Imaging

Radiography is a first-line diagnostic tool in routine HO detection. The most commonly used HO classification systems, such as the Brooker classification for the hip and Hastings and Graham classification of the elbow, are based on the X-ray assessment [3]. Computed tomography (CT) can provide a more accurate assessment of the relation of HO lesion to the joint and other vascular and neural structures. CT and X-ray examinations remain the gold standard in the imaging diagnosis due to a low cost, simplicity, and high effectiveness in detecting fully developed HO lesions [52]. Nuclear medicine modalities can also be useful and provide metabolic and functional information on developing HO. The scintigraphy, including planar bone scan and single-photon emission computed tomography (SPECT), is proven to be a highly sensitive method in HO detection [53,54]. Similarly, the positron emission tomography (PET) can be useful in the HO diagnosis and successfully identify early HO and chronic lesions [55]. The other diagnostic imaging techniques include magnetic resonance imaging (MRI) that can identify vascularization and increased density in the early phases of HO as early as two days after the onset of clinical symptoms [38]. Recently, the ultrasonography is gaining popularity in HO detection and monitoring due to its safety profile, low cost, and the possibility of bedside-application [56]. In diagnostic imaging, it is critical to distinguish HO from neoplastic processes such as osteosarcoma, deep venous thrombosis (DVT). HO can also mimic gout, avulsion fracture, or local tissue calcifications like dystrophic and tumoral calcification or calcific tendonitis [11,52].

### 2.6. Biomarkers

The serum alkaline phosphatase enzyme (ALP) was extensively investigated as a potential biomarker of HO in traumatic HO, NHO after spinal cord injury, and total hip replacement induced HO. The elevated ALP level reflects enhanced bone turnover and increases with osteoclast activity. Detection of ALP could serve as a relatively inexpensive and widely available test for HO. Serum ALP concentration increases about two weeks after the operation reaching the peak concentration at week 10–12 and returns to the base level at week 18. However, ALP levels can be normal in the presence of HO development (55.2%), and the usefulness of ALP in HO screening is being discussed. Similarly, a bone-specific isoform of alkaline phosphatase (BAP) can be elevated in HO patients, but BAP levels are normal in most cases (67.8%). The other tested HO biomarkers are urinary excretion of type I collagen cross-linked C-telopeptide (CTX-1) and prostaglandin E2 (PGE2). The classical inflammation marker C-reactive protein, CRP is elevated in 77.0% of HO patients, but it is not specific [39,57,58]. Additionally, cytokine levels are investigated as biomarkers of HO onset. In the mouse model of FOP, the level of monocyte chemoattractant protein 1 (MCP1) (serum, saliva), IL1β (saliva), and tumor necrosis factor α (TNFα) (serum) were significantly increased compared to control group. In the mouse model of trauma-induced HO, the levels of TNFα, IL1β, IL6, and MCP1 were increased in serum samples [59]. In human studies, HO was associated with the level of serum (IL3, IL12) and wound effluent cytokines (IL3, IL13) in combat-injured patients [60].

Recently proteomic biomarkers were analyzed with mass spectrometry in non-genetic HO patients. Significant differences were found in the levels of certain peptides in patients with HO compared to the non-HO group. The researchers point out the protein fragments of osteocalcin (OC), collagen alpha 1 (COL1), osteomodulin (OMD) as potential clinical biomarkers for HO [61]. Another investigated class of HO biomarkers are small non-coding RNA molecules (miRNA). The disregulation of miRNA homeostasis may play a vital role in HO development. For instance, the decreased expression of miRNA-630, which is responsible for endothelial cells transition towards mesenchymal cells, was observed in HO patients [62]. The decreased level of miRNA-421 in humeral fracture patients is associated with BMP2 overexpression and a higher rate of the HO occurrence [63]. The miRNA-203 downregulation leads to an increase in expression of runt-related transcription factor 2 (Runx2), which is a crucial osteoblast differentiation regulator [64]. The miRNA particles are not only possible HO indicators, but they can also be future therapeutic targets.

### 2.7. Prophylaxis

The standard HO prophylaxis is pharmacological treatment with nonsteroidal anti-inflammatory drugs (NSAIDs) or local external beam radiotherapy (RT). The NSAIDs or radiotherapy prophylaxis is a well-proven and effective method, but it is not specific. Currently, the more targeted pharmacological strategies are being tested and developed for inhibiting specific pathways and molecules responsible for HO. Once the mature lesion is developed, it is not possible to reverse the changes, and the only remaining treatment option is surgical resection [65]. Despite that NSAIDs are effective prophylaxis of HO, they do not present efficacy when HO is fully developed. There is no difference between non-selective NSAIDs and selective NSAIDs in HO treatment [66]. The selective cyclooxygenase-2 (COX-2) inhibitors can significantly decrease discontinuation of treatment due to gastro-intestinal (GI) side effects [67,68]. However, non-selective NSAIDs are the most commonly used in clinical practice (87%) and remain “golden standard” in HO prevention. Indomethacin non-selective COX inhibitor is the most commonly prescribed NSAID for HO prophylaxis (57%) with a daily dose of 100–150 mg with a mean of 30 days of administration [69,70]. In addition to high efficiency, NSAIDs are approximately 45 times more cost-effective compared to RT [71,72].

RT recommended before the surgery or early, up to 72 h post-surgery, is an equally successful method for prophylaxis of HO development as NSAIDs. The multiple fractions RT is more effective in the reduction of HO. It is dose-dependent, but a modification of a biologically effective radiation dose over the >2500 cGy did not result in better effectiveness [73]. In total hip replacement patients, the combination of NSAIDs and RT may also be beneficial [74]. The RT seems to be a safe method of HO prevention in total hip replacement patients regarding local neoplastic processes and aseptic loosening of the implant [75].

The other prophylaxis modalities were also proposed. Taking into account bacterial contamination of wound in traumatic HO, locally administered vancomycin prophylaxis suppressed HO in trauma-induced rats infected with methicillin—resistant *Staphylococcus aureus* (MRSA) [76]. Bisphosphonates that are mainly used as anti-osteoporosis drugs and act by inhibiting calcification, and bone resorption dependent on osteoclasts, have no significantly higher efficacy than NSAIDs [77]. The aspirin, which has both effects of NSAID and the anti-platelet agents, is often used for venous thromboembolism (VTE) prophylaxis in total hip replacement patients and was also shown to be effective in the HO rate reduction [78].

### 2.8. Treatment

Surgical removal of lesions is currently the only effective method when HO is already formed and gives clinical symptoms. However, the operation itself may induce the formation of new ossifications. Among the indications of HO are pain and reduction of ROM. In most cases, the treatment also includes NSAIDs or radiotherapy as the prevention of relapse. A common strategy is to change the type of prophylaxis or the application of another type of NSAIDs class if the previously used prophylaxis has failed. The standard procedure is simple excision of HO, but it is unclear whether it should be removed completely or only partially [79]. Some authors recommend HO surgery only when the mature bone tissue is formed. However, early intervention minimizes the development of intra-articular changes and HO recurrence, so ossifications should be removed as soon as the mature bone is formed, without unnecessary delay [80,81,82,83]. As a result of surgery, the pain level is reduced and ROM increases, which significantly improves the function and often reduces the level of pain [70,84,85,86,87,88]. The total hip replacement is a promising solution for NHO in the area of the hip joint in patients after traumatic brain injury. The standard procedure is the Girdlestone procedure, but total hip replacement seems to give better results than a simple excision. When using THA, ossification has less tendency to relapse and the patient achieves more satisfactory functional results. [89,90].

## 3. Heterotopic Ossification Precursor Cells

### 3.1. Stem and Progenitor Cells in Skeletal Muscles

**Figure 1:**
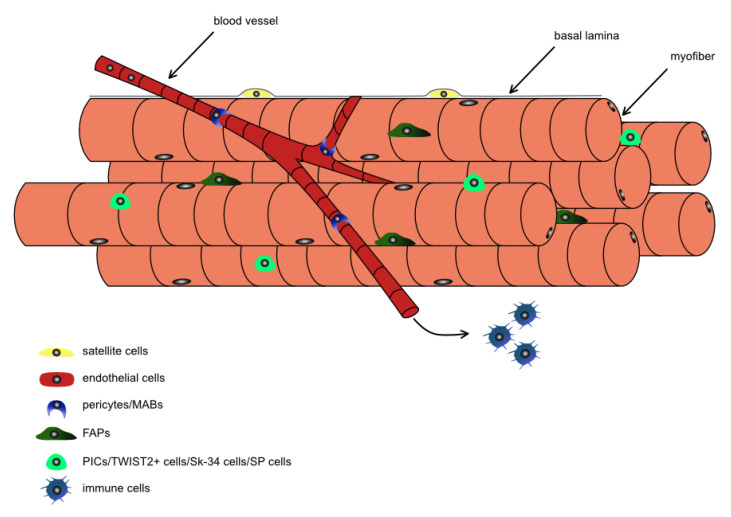
The stem and progenitor cells responsible for skeletal muscle homeostasis. The multinucleated skeletal muscle myofibers are accompanied by several types of stem and progenitor cells, such as satellite cells, endothelial cells, pericytes, mesoangioblasts (MABs), and fibro-adipogenic progenitors (FAPs), which could participate in regeneration. Other populations of muscle interstitial cells, such as, PW1+/PAX7 interstitial cells (PICs), Sk-34 cells, TWIST2+ cells, side population (SP) cells was also shown to be able to follow myogenic program. Moreover, the skeletal muscle reconstruction is accompanied by infiltration by immune cells.

HO development is a complex process engaging many different cell types. Several lines of evidence suggest that the development of HO in skeletal muscle could be a result of pathological differentiation of stem and progenitor cells present in skeletal muscle. The most important cells responsible for postnatal skeletal muscle growth and regeneration are satellite cells (SCs), i.e., unipotent stem cells located between muscle fibers plasmalemma and basal lamina (Figure 1). These cells are activated in response to skeletal muscle injury which results in the cell cycle re-entry [91]. The signals activating satellite cells are provided by damaged muscle fibers, inflammatory cells, and endothelium [92]. Activated SCs start to proliferate, differentiate into myoblasts, i.e., muscle progenitor cells, and then myocytes. The myocytes fuse with existing myofibers or with each other to form myotubes and then, after innervation, myofibers. Many studies showed that SC presence is essential for skeletal muscle regeneration [93]. This multi-step process is accompanied by changes in expression of pair box transcription factors 7 (Pax7) and myogenic regulatory factors (MRFs), such as MYOD, MRF5, myogenin, MRF4, as well as skeletal muscle structural proteins [94]. Importantly, SCs are able to follow two different fates—they could maintain PAX7 and down-regulate MYOD expression to self-renew their population or down-regulate PAX7 and maintain MYOD expression to upregulate MYOGENIN and initiate differentiation [94]. SCs proliferation is regulated by MYOD and MYF5 which control the activity of the genes involved in DNA replication and cell cycle progression, such as cell division cycle 6 protein (CDC6) and minichromosome maintenance complex component 2 (MCM2). MYOD contribution in the myogenic differentiation also involves the induction of miR206 and miR486 which downregulate PAX7 [95]. Moreover, long non-coding RNA linc-RAM promotes the formation of MYOD complex with chromatin modifier BAF60c which enables MYOD binding to promoters of target genes and marks the chromatin for recruitment of chromatin-remodeling complex, i.e., BRG1-based SWItch/Sucrose NonFermentable (SWI/SNF). This MYOD-BAF60c-BRG1 complex remodels the chromatin and activates transcription of MYOD-target genes [96]. Furthermore, MYOD, as stated above, promotes expression of MYOGENIN and MRF4, i.e., transcription factors responsible for myoblast cell cycle exit and their differentiation into myocytes and myotubes. These differentiation steps are accompanied with expression of myosin heavy chains (MHC), enolase 3 (ENO3), and muscle creatine kinase (MCK) [91].

Importantly, the fate of SCs is determined by their interactions with the niche. The quiescent SC niche is formed by myofibers and the extracellular matrix (ECM), i.e., the basal lamina. Such a niche is modified after the skeletal muscle injury and during regeneration. The factors secreted by damaged myofibers, inflammatory cells, endothelial cells, fibroblasts, and fibro-adipogenic progenitors (FAP), present in skeletal muscle, regulate the fate of SCs and myoblasts. Since the inflammation is among the initial responses to muscle injury, resident immune cells, such as mast cells and neutrophils, are activated by factors released by degenerated fibers [97]. The immune cells start to produce pro-inflammatory molecules, such as histamine, TNFα, interferon γ (IFNγ), IL1β, which leads to increased vascular permeability and myeloid cells recruitment. Both neutrophils and macrophages participate in damaged myofibers removal. Simultaneously, factors secreted by neutrophils and macrophages play an important role in the SC activation and myoblast proliferation and differentiation. Thus, the depletion of macrophages reduces the level of hepatocyte growth factor (HGF) and insulin-like growth factor 1 (IGF1), causing impairment of skeletal muscle regeneration [98]. HGF binds with c-met and plays a role in SC activation [99]. IGF-1 promotes myoblasts proliferation and differentiation [100,101]. Macrophages also secrete TNFα and IL6, i.e., factors which promote myoblasts proliferation and differentiation [102]. Other cells that play crucial role in skeletal muscle reconstruction are endothelial cells. They participate in the restoration of vasculature in damaged muscle and secrete pro-angiogenic and pro-myogenic factors, such as apelin, oncostatin, and periostin [103,104,105]. ECM remodeling that is an important step during muscle regeneration involves fibroblasts and FAPs (also named “mesenchymal progenitors”). These cells also produce pro-myogenic factors, such as: IGF1, IL6, and follistatin [106,107,108]. FAPs are interstitial non-myogenic progenitors expressing platelet derived growth factor receptor α (PDGFRα) [109,110,111]. In intact muscle FAPs are quiescent but after an injury they start to proliferate and synthesize ECM proteins, as well as abovementioned factors [112]. In aged muscles and during chronic diseases the FAPs accumulation and differentiation into fibroblasts and adipocytes is observed. Thus, these cells could be engaged in the formation of fibrosis or adipose tissue accumulation [112].

Except for abovementioned cell populations, skeletal muscle interstitium is the source of stem and progenitor cells different form SCs [113]. Their role in skeletal muscle homeostasis is extensively studied using mouse models. However, many studies also focus on human cells [113]. In mouse as well as in human muscles pericytes and mesangioblast are localized peripherally to microvessel endothelium. They are described as PDGFRβ, NG2, CD146 expressing cells [114,115,116,117,118,119]. Such cells were shown to be able to fuse with myofibers and occupy satellite cell niche in regenerating muscle [114,115,116,117,118]. Moreover, mouse and human pericytes secrete IFG-1 and angiopoetin that are known factors supporting myoblasts differentiation [120]. Other populations detected in mouse muscles are PW1+/PAX7 interstitial cells, i.e., PICs expressing PW1, SCA1, and CD34 [121]. These cells transplanted to injured mouse muscles participated in the regeneration and restoration of SC population [121]. Next, the TWIST2+ progenitor cells expressing transcription factor TWIST2, myoendothelial cells expressing CD34, i.e., Sk34 cells, and side population (SP) cells isolated on the basis of Hoechst day exclusion were identified in mouse muscle interstitium [122,123,124]. They showed myogenic potential in vitro and formed new myofibers after transplantation into injured muscles [122,123,124], similarly to CD133+ cells presented in human muscles [125].

### 3.2. The Osteogenic Potential of Stem and Progenitor Cells Residing in Skeletal Muscle—In Vitro Studies

Few populations of stem and progenitor cells residing in skeletal muscle and described above could follow osteogenic differentiation in vitro. Among them are mouse and human SCs. BMP4 and BMP7 treatment of mouse SCs induced their osteogenic differentiation, which was shown by increased expression of ALP (and also its activity), osteopontin, and osteocalcin, i.e., the markers of osteogenic differentiation. Moreover, SCs were able to undergo spontaneous osteogenic differentiation when cultured in Matrigel [126]. Osteogenic properties were also documented for human SCs after their in vitro culture in osteogenic differentiation medium (OB-1, ZenBio). After 14 days of treatment, cells increased expression of osteogenic differentiation genes, such as, RUNX2 and BGLAP. Moreover, Alizarin Red staining revealed the accumulation of calcium deposits [127]. Further, such staining of mouse skeletal muscle-derived TBX18+ pericytes, cultured in medium supplemented with dexamethasone, L-ascorbic acid-phosphate, β-glycerophosphate, and BMP2, revealed the deposition of mineralized matrix also indicating differentiation in osteogenic lineages [128]. Similarly, human ALP+ pericytes were able to differentiate in osteoblasts after BMP2 treatment in vitro [117]. On the other hand, CD146+/ALP+ progenitors isolated from human skeletal muscles were not able to follow osteogenic program in vivo after transplantation with hydroxyapatite/tricalcium phosphate scaffold [129]. Finally, mouse FAPs characterized by the presence of markers such as TIE2, PDGFRα or SCA1 differentiated into osteoblasts formation after BMP7, BMP2, treatment or when cells were cultured in osteogenic differentiation medium containing dexamethasone, β-glycerophosphate, and ascorbic-acid [109,110,130]. So far, osteogenic differentiation has not been analyzed or documented for other cell populations, such as PIC, TWIST2+ cell, Sk34 cells, as well as human circulating CD133+ cells [121,123,125,131,132,133,134,135,136].

### 3.3. The Cells Directly Participating in Heterotropic Ossification Formation In Vivo

Different animal models, which could be divided into two groups, were used to follow the cells responsible for HO development [1,5,21]. The first one consists of genetically modified animals, i.e., mouse engineered to express, in controlled manner, constitutively active ACVR1, which mimic FOP. The second group includes animal models in that trauma was caused by muscle blunt-force or forced ROM damage, muscle dissection, hip surgery or skin burn with Achilles tenotomy. The third group includes animals in which HO develops after BMPs injection or overexpression. The fourth model bases at the spinal cord injury in conjunction with cardiotoxin induced muscle damage. Moreover, a lot of information about the cell types responsible for HO formation was obtained thanks to lineage tracing [1,5,21].

Using the abovementioned models, a few cell populations were designated to be responsible for HO formation. As mentioned, several skeletal muscle cell types, such as human pericytes and mouse SCs, and FAPs present osteogenic potential in vitro. Importantly, in vitro results cannot be directly translated to in vivo situation. Notably, in vivo studies using mouse models proposed that HO precursors could originate from skeletal muscle endothelial, “mesenchymal” or pericyte populations or tendon and connective tissue cells or even circulating stem/precursor cells [1,5,21,137]. Some initial studies suggested that endothelial cells, characterized by the presence of TIE2, which is the tyrosine kinase receptor for angiopoetin, are engaged in HO formation [138,139]. Tracing these cells on the basis of Tie2 expression proved that they participate in HO development after BMP2 intramuscular injection or cardiotoxin induced skeletal muscle injury in transgenic mice that overexpressed BMP4 at neuromuscular junction [138,139]. Importantly, neither SCs (expressing *MyoD*) nor vascular smooth muscle cells (expressing smooth muscle myosin heavy chain) contributed to HO [138]. Moreover, also other lineage tracing and transplantation experiments clearly showed that SCs did not participate in HO development [130,138,140]. The presence of TIE2 expressing cells was observed in human fragments of tissue from FOP patients [139]. However, the studies in which the cells expressing *Cdh5* (VE-cadherin), i.e., endothelial progenitor cells, were traced, showed that in HO lesion such cells were located only peripherally [141]. Thus, the endothelial progenitors did not participate in HO formation [141]. Moreover, it was showed that TIE2 is not unique marker of endothelial cells. It is also expressed by mouse muscle interstitial cells that are able to follow osteogenic program. Next, TIE2+ cells express PDGFRα and SCA1 and do not express CD31 and CD45 [130]. Thus, these cells correspond to the population of FAPs described in human and mouse skeletal muscles [109,142]. The mesodermal origin of HO precursors was also proven by tracing of PRX1+ cells after tenotomy resulting in the formation of HO [143,144]. During embryogenesis *Prx1* gene is expressed in tissues of mesodermal origin and is crucial for cartilage and bone development. *Dermo1* gene expression, on the other hand, is restricted to the perichondrium. Tracing of DERMO+1 cells showed their engagement in HO development [143]. The localization in skeletal muscle interstitium was also demonstrated for MX1 expressing cells that form HO in response to muscle injury in mice expressing constitutively active form of ACVR1 [145]. MX1 is interferon induced GTP binding protein and is expressed in skeletal muscle interstitial cells, bone marrow osteoprogenitors and endothelial cells [145]. Lineage tracing method allowed further characterization of HO precursor cells. Thus, it was shown that GLAST or GLI1 expressing cells form HO [146,147]. GLAST, i.e., glial high affinity glutamate transporter, is expressed in different tissues and among them are interstitial cells of connective tissue and pericytes [147]. GLI1, i.e., glioma-associated oncogene 1, is a transcription factor engaged in HEDGEHOG signaling [146]. In the skeletal muscle interstitium *Glast* or *Gli1* expressing cells were localized close to vasculature, co-expressed fibroblast-specific protein 1 (FSP1), STRO1, and PDGFRα [146,147]. On the other hand, it was also well documented that NG2+ pericytes, similarly to endothelial or hematopoietic cells, did not participate in HO development [145].

The other source of HO precursors is tendon and connective tissue within the skeletal muscle [145,148]. The cells expressing transcription factor scleraxis (*Scx*) were localized in the tendon and ligaments [145]. However, the presence of *Scx* expressing cells was also noticed in the connective tissue within the skeletal muscle [148]. The SCX+ cells also express PDGFRα, SCA1, and S100A4 [148]. *Scx* expressing cells are able to develop HO localized in the tendon and joints spontaneously in mice expressing constitutively active form of ACVR1 and after tendon injury or intramuscular loading of BMP containing-scaffold [145]. In such mice, the HO developed only in injured muscles [148].

Summarizing, the question about the HO precursor cell identity is still open. Evidence presented above allowed us to conclude that potential HO precursors are of mesodermal origin and are located in the skeletal muscle interstitium. In mouse cells able to form HO could be identified on the basis of TIE2, PDGFRα, SCA1, GLAST, FSP1, STRO1, GLI1, and MX1 expression. Moreover, such cells should show many similar features to skeletal muscle FAPs and pericytes. Tendon and connective tissue present within skeletal muscle could be considered as the source of cells responsible for HO formation. In human, however, such cells are not precisely described yet. Moreover, it is also suggested that different types of cells could be responsible for HO development dependently of HO type [1,5,21,137].

## 4. Possible Signaling Mechanisms of Ectopic Osteogenesis in Skeletal Muscles

The knowledge on molecular mechanisms of HO formation is limited. It is well established that ectopic osteogenesis occurs as a result of traumatic injury, severe burns, and is commonly observed after invasive surgeries, which indicates that it is related to inflammation. However, the precise immune and signaling regulation is poorly understood. One of the best-known regulators of bone development and postnatal bone maintenance are bone morphogenic proteins (BMPs) [149]. BMPs are members of TGFβ superfamily, which also consists of TGFβ, activins or inhibins. Canonical TGFβ/BMP signaling is a linear cascade which involves TGFβ/BMP ligands, two types of receptors (type I and II), and signal transducers—SMADs. Receptor binding to BMP leads to SMADs—SMAD1/5/8, to TGFβ leads to SMAD2/3 phosphorylation. Activated SMADs bind to SMAD4, then the complex is accumulated in nucleus where regulates target gene expression [150]. One of the downstream targets of these pathways is for example gene encoding RUNX2, well-known master regulator of osteogenesis which is also aberrantly expressed in the ossified soft tissues [151,152,153,154]. TGFβ dependent activation of SMAD2/3 promotes osteoprogenitors migration and early stages of differentiation, while negatively regulates further steps of osteogenesis. SMAD2/3 phosphorylation inhibits RUNX2 expression and activated SMAD3 recruits class II histone deacetylases (HDACs) 4 and 5 which inhibit RUNX2 function. Although TGFβ-SMAD3 negatively regulates osteoblastogenesis, it also inhibits osteoblast apoptosis and differentiation into osteocytes [155]. On the other hand, there is TGFβ dependent non-SMAD pathway which also contributes to bone formation. TGFβ binding to its receptors can result in activation of MAPK p38 or MAPK ERK1/2 pathways through TAB1-TAK1 complex which leads to positive regulation of RUNX2 activity and favors osteoclast differentiation [156]. It indicates that TGFβ molecule is coupling bone formation, through RUNX2 phosphorylation, osteoprogenitors enrichment or osteoblast proliferation promotion, and inhibition of apoptosis with bone resorption through inhibition of RUNX2 expression and function and osteoclast maturation [157]. BMPs binding to receptor leads to SMAD1/5/8 phosphorylation (except BMP3 which action leads to SMAD2/3 phosphorylation). Activated SMAD1/5/8 bind with SMAD4 and promote expression of many osteogenesis promoting factors like RUNX2, OSX or DLX5. Similarly, to TGFβ, BMPs also can activate SMAD-independent pathway through phosphorylation of TAK1-TAB1 complex and activation of MAPK p38 or MAPK ERK1/2 pathways. In conclusion, most BMP ligands are strong osteogenic agents, acting through both SMAD-dependent and SMAD-independent signaling pathway, which synergize osteogenic transcriptional factors like RUNX2 or OSX [157,158].

In vitro and in vivo exogenous stimulation of TGFβ/BMP signaling (BMP2, BMP4, BMP9 or TGFβ) is commonly used for induction of ossification. Those proteins, especially BMP2 and BMP9, are also highly expressed in human HO [159]. One of the most intensively studied disease which manifests itself in severe HO is FOP. It is still unclear, however, what the cellular and molecular mechanisms are that cause pathological effects. Analyzes of human mesenchymal stromal cells (MSC; expressing CD44, CD73 and CD105), derived from induced pluripotent stem cells (iPSC) obtained from FOP which patients, showed that these cells were characterized by higher activity ofof SMAD1/3/5, SMAD2/3 and MAPK ERK1/2 when compared to genetically corrected resFOP-iPSCs-MSCs [160]. Most studies suggest that mutation in ACVR1 present in FOP patients cells causes hypersensitivity to BMPs, which results in constitutive phosphorylation of its receptor, and continuous signal transduction via phosphorylation of SMAD1/5/8. As a result downstream targets of BMP signaling, like ID-1, OSX or RUNX2 are expressed [130,161,162]. However, recent report, based on study of murine FAPs, demonstrated that R206H substitution in ACVR1 may be neomorphic and altering signaling specificity to activins. Normally, activins binding to ACVR1 receptor lead to SMAD2/3 phosphorylation. Obtained results suggest that Activin A binding to mutated ACVR1 (R206H) receptor leads to SMAD1/5/8 instead of SMAD2/3 phosphorylation which results in ectopic bone formation [163]. It is well established that TGFβ/BMP signaling crosstalks with other pathways during embryonic and postnatal development, similarly as with MAPK described above. For example, crosstalk between canonical WNT pathway, TLR pathway or mTOR pathway was described [164]. TLR signaling intermediate evolutionary conserved signaling intermediate in toll pathway (ECSIT) is necessary for BMP signaling in formation of mesoderm during mouse embryogenesis [165]. Additionally, β-catenin was shown to be necessary for bone development and osteoblast formation in mouse embryos [151]. Other studies showed that β-catenin complexed with T cell factor 1 (TCF1) directly stimulates Runx2 expression [166]. Another study suggested that β-catenin, together with other proteins like SMAD1, DLX5, Sp7 or SOX6, form an enhanceosome which binds to enhancer of Runx2 gene and promotes its expression [167].

**Figure 2:**
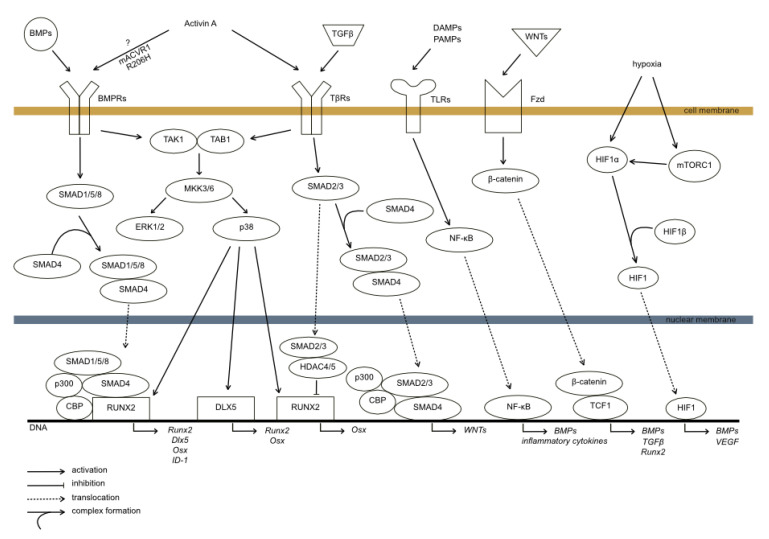
Possible signaling mechanisms of ectopic osteogenesis in skeletal muscles. BMPs bind to homomeric type II receptors which phosphorylate homomeric type I receptor and induce SMAD-dependent and SMAD-independent signaling. In the SMAD-dependent signaling SMADs 1, 5 or 8 complex with SMAD4 and translocate to the nucleus where recruit RUNX2 and other co-factors to regulate osteogenic gene expression. TGFβ binds to complex of two TGFβ types I receptors (TβRI) and two type II receptors (TβRII), which phosphorylate each other and induce SMAD-dependent and SMAD-independent signaling. In the SMAD-dependent signaling activated SMAD2/3 form complex with SMAD4. Complex translocates to the nucleus where recruits co-factors and regulates target gene expression. Activated SMAD3 recruits HDACs which inhbit RUN2 activity. In the SMAD-independent pathway, regardless of the ligand bind to the receptors, TAK1 recruits TAB1 to initiate p38 MAPK or ERK1/2 MAPK signaling cascade. MAPK phosphorylates and activates RUNX2, DLX5, and OSX transcription factors. Activation of TLR singaling pathways by PAMPs and DAMPs lead to activation of nuclear factor-kappaB (NF-κB), which controls the expression of an array of inflammatory cytokine genes and BMPs. WNTs bind to Frizzled (Fzd) receptors and activate the canonical WNT pathway which leads to accumulation of β-catenin in the cytoplasm. β-catenin is translocated to the nucleus where forms complex with TCF1 which acts as transcriptional activator of *Runx2*. Low level of oxygen (hypoxia) induces the mTOR pathway. HIF1α, a downstream intermediate in mTOR signaling, is a key transcriptional regulator of the cellular response to hypoxia. It forms complex with HIF1β and as HIF1 enters to the nuclei where regulates target gene expression.

Hypoxia and inflammation are also among the factors implicated in the episodic induction of ectopic bone formation. Notably, mTOR modulates hypoxic and inflammatory signaling during the early stages of HO. At the later stages of HO, however, mTOR signaling is critical for chondrogenesis and osteogenesis [164]. Increase in mTOR signaling was shown using mouse model of FOP, i.e., animals which express constitutively active activin receptor, i.e., ACVR1 [168] and its inhibitor, rapamycin, has been shown to suppress HO formation [168]. Hypoxic environment stabilizes hypoxia-inducible factor 1α (HIF1α) which regulates expression of many proteins, such as VEGF or BMPs, which are involved in HO formation [169]. Analysis of three different mouse FOP/HO models have demonstrated hypoxia and increased HIF1α signaling [144]. Expression of HIF1α was also increased in adipose samples derived from severely burned patients, i.e., those ones being at risk for trauma-induced HO development [144,170]. Interestingly, analysis of human HO tissues, human preosteoblasts (hFOB1.19) and tissues of mice serving as a model of HO, revealed that miRNAs have essential role in osteoblast differentiation and HO development. miRNA-203 have been shown to be negatively correlated with HO and to participate in inhibition of osteoblast differentiation by directly binding to RUNX2 [64]. Nevertheless, the mechanisms underlying the development of HO in patients that not carry any mutations are still obscure. Moreover, even in FOP patients bone formation is not always observed in their soft tissues. Bone formation seems to be rather a result of injuries and inflammation, which strongly suggests a link between immune response and HO. In vivo studies using rabbits showed that bacterial transplantation into tibia bone increased inflammation-driven bone formation. In the same study, lipoteichoic acid (LTA)—the bacterial cell-wall derived toll-like receptor 2 (TLR2) activator—was identified as an osteo-stimulatory factor [171]. Other studies involving FOP patients-derived connective tissue progenitor cells revealed that such cells present much higher expression of TLRs in comparison to cells that are expressing normal ACVR1 receptor. That effect was even more significant after TNFα treatment of examined cells. The same study also revealed that TLR signaling can induce SMAD1/5/8 phosphorylation. Additionally, ECSIT, complex including TAK1 and TRAF6, which plays pivotal role in TLR-mediated NK-kB and SMAD1/5/8 signaling, was identified as a link between TLR-pathway and BMP pathway in human FOP-connective tissue progenitor cells [172,173]. As described above, normal cells, not carrying any mutations in *ACVR1*, can undergo osteogenesis after BMP stimulation. Thus, development of heterotopic bone provides the signaling environment in which BMP level is sufficient enough to stimulate the cells to form the bone [163]. Results of these reports together suggest that main mechanism of HO formation is connected with TGFβ/BMP signaling, especially SMAD1/5/8 action, which leads to expression of osteogenic transcription factors. Factors present in damaged tissue lead also to activation of mTOR, WNT or TLR pathways which may cross-talk with TGFβ/BMP or independently promote osteogenic factors expression and induce HO formation. Even small ossification within tissue provide a BMP rich environment, which further stimulates neighboring cells to follow osteogenic differentiation and support newly creating bone growth. However, it still remains unclear why spontaneous HO is observed or how that process is induced and regulated (Figure 2).

## 5. Future Therapeutic Options

Currently, a clinical trial phase 3 of highly specific retinoic acid receptor γ (RARγ) agonist, R667 (palovarotene), carried out by Clementia Pharmaceuticals involves 90 FOP patients (NCT03312634). RAR is a strong inhibitor of chondrogenesis. Stimulation of its γ subtype reduces in BMP signaling by lowering SMAD1/5/8 phosphorylation and as a result decreases HO formation. The safety profile of this drug is being carefully assessed due to the teratogenic potential of RAR agonists and other side effects, including cheilitis, xerosis, dryness of mucous membranes, inhibition of growth plates in children, hearing and vision impairment [70,174]. Another investigated strategy is blocking mutant ACVR1. The ACRV1 stimulates BMP through SMAD1/5/8 signaling and promote HO. Such approaches involve anti-Activin A antibody (REGN2477) and which is currently at phase 2 of randomized control trial for 44 FOP patients (NCT03188666, Regeneron Pharmaceuticals) [175]. Other ACVR1 direct inhibitors (AZD0530 and PD 161570) are being investigated. The AZD0530 difumarate inhibits both BMP and TGFβ signaling. Phase 2 study involving AZD0530 (Saracatinib) to prevent FOP is currently carried out by VU University Medical Center (NCT04307953) [176]. The other treatment strategies include a local application of apyrase, which influences the BMP-SMAD pathway by the reduction of SMAD1/5/8 phosphorylation [177]. The BMP receptor antagonists, such as noggin, also inhibit HO in animal models [178]. Researchers also suggest that pharmacological inhibition of HIF1α using PX-478, rapamycin, apigenin, or imatinib can reduce pathologic extraskeletal bone formation [144,179]. Recently, gene therapy opportunities raised for HO. Non-virus-mediated transfer of small interfering RNA (siRNA) particles against mRNA encoding *Runx2* and *Smad4* inhibited HO in rats after Achilles tenotomy [180]. The siRNA could also possibly directly block mutant ACVR1 as a therapeutic option in future studies [179]. Additionally, the immune system may be a potential target for HO prevention. Neutralizing antibodies against immune checkpoint proteins (ICs) block limit the extent of HO in animal studies [9].

## 6. Conclusions

In this review we summarize current knowledge on the development of different forms of HO. Numerous projects involving analysis of patients’ tissues and also animal models allowed great advancement in the understanding of this pathology. However, we are still not certain about the precise sources of the osteogenic progenitors involved in this pathology. Additionally, the knowledge on the signaling pathways deregulated despite being enormous is still not sufficient to design the properly targeted treatment. Nevertheless, what we already know allowed us to propose several hope-giving therapeutic approaches which are currently tested. Thus, more work needs to be done, but it seems that we are on the proper path.

## Abbreviations

ACVR1/ALK2	Activin A receptor type 1/activin-like kinase 2
ALP	alkaline phosphatase
BAF60c	60 KDa BRG-1/Brm-Associated Factor Subunit C
BAP	bone-specific isoform of alkaline phosphatase
BMP	bone morphogenetic protein
CD	cluster of differentiation
CDC6	cell division cycle 6 protein
COL-1	collagen alpha 1
COX-2	cyclooxygenase-2
CRP	C-reactive protein
CTDA	cervical total disc arthroplasty
CTX-1	type I collagen cross-linked C-telopeptide
Dlx5	Distal-Less Homeobox 5
DVT	deep venous thrombosis
ECM	extracellular matrix
ENO3	enolase 3
ERK	extracellular signal-regulated kinase
FAP	fibro-adipogenic progenitors
FOP	fibrodysplasia ossificans progressive
FSP1	fibroblast-specific protein 1
GLAST	glial high affinity glutamate transporter
GLI1	glioma-associated oncogene 1
GNAS	guanine nucleotide-binding protein, subunit alpha
HDAC	histone deacetylase
HGF	hepatocyte growth factor
HIF1α	hypoxia-inducible factor 1α
HLA	human leukocyte antigen
HO	heterotopic ossification
ID-1	DNA-Binding Protein Inhibitor ID-1
IFN γ	interferon γ
IFOPA	international FOP Association
IGF1	insulin-like growth factor 1
IL	Interleukin
iPSC	induced pluripotent stem cell
LTA	lipoteichoic acid
MAPK	mitogen-activated protein kinases
MCK	muscle creatine kinase
MCM2	minichromosome maintenance complex component 2
MCP-1	monocyte chemoattractant protein-1
MHC	myosin heavy chains
miRNA	microRNA
MIS	micro-invasive surgery
MRF	myogenic regulatory factor
MRI	magnetic resonance imaging
MRSA	methicillin-resistant Staphylococcus aureus
MSC	mesenchymal stromal cell
mTOR	mammalian target of rapamycin
NF-κB	Nuclear factor-κB
NG2	neural-glial antigen 2
NHO	neurogenic heterotopic ossification
NSAID	nonsteroidal anti-inflammatory drug
NT-3	neurotrophin-3
OC/BGLAP	osteocalcin/Bone Gamma-Carboxyglutamate Protein
OMD	Osteomodulin
OSX/Sp7	Osterix
Pax7	paired box transcription factor 7
PDGFRα	platelet derived growth factor receptor α
PGE2	prostaglandin E2
PIC	PW1 interstitial cell
POH	progressive osseous heteroplasia
Prx1	peroxiredoxin Prx1
ROM	range of motion
RT	Radiotherapy
RUNX2	runt-related transcription factor 2
SC	satellite cell
SCA1	stem cell antigen 1
SCI	spinal cord injury
Scx	Scleraxis
Sox6	SRY-Box Transcription Factor 6
SP cell	side population cell
SPECT	single-photon emission computed tomography
SWI/SNF	SWItch/Sucrose NonFermentable
TAB1	TAK1 binding protein
TBI	traumatic brain injury
TBX18	T-box transcription factor 18
TCF1	T cell factor 1
TGFβ	transforming growth factor β
THR	total hip replacement
TIE2	angiopoietin receptor
TLR	toll-like receptor
TNF-α	tumor necrosis factor-α
TRAF6	TNF Receptor Associated Factor 6
TWIST2	Twist Basic Helix-Loop-Helix Transcription Factor 2/DERMO1
VE-cadherin	vascular endothelial cadherin (Cdh15)
VEGF	vascular endothelial growth factor
VTE	venous thromboembolism
WNT	wingless/integrated
